# Scaling sports equipment for children promotes functional movement variability

**DOI:** 10.1038/s41598-020-59475-5

**Published:** 2020-02-20

**Authors:** Tim Buszard, Alessandro Garofolini, Machar Reid, Damian Farrow, Luca Oppici, David Whiteside

**Affiliations:** 1 Institute for Health and Sport (IHES), Victoria University, Victoria, Australia; 2 Game Insight Group, Tennis Australia, Melbourne, Australia; 3 Psychology of Learning and Instruction, Department of Psychology, School of Science, Technische Universität Dresden, Dresden, Germany; 4 Centre for Tactile Internet with Human-in-the-Loop (CeTI), Technische Universität Dresden, Dresden, Germany

**Keywords:** Medical research, Paediatric research

## Abstract

Scaling sports equipment to match the physical development of children allows motor skills to be performed with greater success and with more desirable movement patterns. It is unknown, however, how scaled equipment affects movement variability – a key factor associated with coordination. Our aim was to identify whether scaled sports equipment facilitates coordination and functional movement variability in children when performing a hitting for accuracy task in tennis. Twenty-five children were asked to execute a forehand stroke with the aim of hitting the ball to a target located 10 metres away. Participants performed the task in two conditions – a scaled equipment condition and a full-sized equipment condition. Scaled equipment led to superior hitting accuracy and greater temporal stability of the swing compared to full-sized equipment. Scaled equipment also afforded the emergence of a functional coupling between upper arm and forearm movement variability which helped regulate the distance between the shoulder and the racket. Comparatively there was a lack of coupling when full-sized equipment was used. Hence, scaled equipment promoted functional movement variability, whereas full-sized equipment resulted in the freezing of mechanical degrees of freedom. This suggests that children’s skill acquisition could be hindered and potentially regress when using inappropriately sized equipment.

## Introduction

The benefits of scaling sports equipment are well documented^1^. By matching the size of equipment to the developmental stage of a player, skills are performed with more success and with more desirable movement patterns^2–7^. This literature, however, is limited by a tendency to focus predominately on performance outcome measures and/or subjective assessments of movement patterns e.g.,^2,3^. It is therefore unclear how equipment affects the coordination of movement with specific reference to functional movement variability – a factor that likely underpins the benefits of scaled equipment for children. The expectation is that appropriately sized equipment facilitates the ability to coordinate movements.

An impressive feature of neurobiological systems is the ability to coordinate multiple joints with abundant degrees of freedom to produce functional movements^8^. According to dynamical systems theory, complex systems – such as a movement system – self organise according to the constraints surrounding the system^9,10^. This self-organisation process allows multiple body parts to coordinate with each other and afford functional movements to emerge. For instance, movements of an individual self-organise based on the confluence of task, individual and environmental constraints^11^. In cricket, reducing the pitch length – a task constraint – resulted in children adopting a bowling technique with less shoulder counter-rotation, which is considered more efficient and less likely to cause injury^12^. Likewise, in tennis, smaller rackets and lower compression balls led to a greater proportion of balls struck out in front of the body and with a low-to-high swing – both of which are considered desirable when playing a forehand stroke^2,3^. Hence, scaling equipment to children’s development is an effective strategy to promote the emergence of desired movement patterns.

Understanding self-organisation is typically accompanied by the measurement of movement variability, which is an inherent feature of the human musculoskeletal system^13^. It has been well documented that movement variability facilitates the self-organisation of functional coordination patterns^14^. In doing so, movements are able to flexibly adapt to the continuous changes of constraints in dynamic environments^9,15^. In hitting and projectile tasks, skilled performers typically display low variability at the end-effector (e.g., the racket, bat, club, or the hand), assuming that each performance occurs at the same location and towards the same target. Yet, proximal elements of the movement system are more variable e.g.,^16–19^. It has been proposed that the proximal segments work to counteract inadvertent variations in one another in order to minimize variability in the end-effector^20^. Coupling body segments whilst achieving low variability in the timing of movements is also a characteristic of skilled performance, as this signifies that the performer can adapt their movements whilst achieving temporal stability which is functional to performance^21^.

This study aimed to identify whether scaled sports equipment facilitated functional movement variability in children when performing a hitting for accuracy task. We opted for a task that emphasised accuracy over speed as sports such as tennis require accuracy, but not necessarily speed, for the game to be played. Functional movement variability was defined as the co-variation between segments in the hitting arm. We hypothesised that scaled equipment, which included a smaller tennis racket and a lower compression ball, would afford the emergence of functional coupling between the upper arm and forearm. More specifically, we expected that the distance between the shoulder and the racket, which represents the length of hitting lever, would be regulated by the coupling of the forearm and upper arm when using scaled equipment. Conversely, when using full-sized equipment, we expected the compensatory coupling to be absent. We expected these results for two reasons: (1) the scaled racket featured a lower moment of inertia and therefore required less force to wield, and; (2) the scaled ball featured less compression, meaning it travelled slower and bounced lower. Consequently, children had more time to strike the scaled ball, and the ball could be struck at a more comfortable height^5^. Additionally, given that scaled equipment was predicted to promote greater success and a functional coupling between segments, we also hypothesised that scaled equipment would lead to greater temporal stability in swing time.

## Results

Hitting accuracy was significantly greater [t (22) = −4.99, p < 0.01, d = 0.75] in the scaled equipment condition than the full-sized equipment condition. The scaled condition also elicited greater temporal stability, as variability in swing time was significantly lower [t (22) = 2.51, p = 0.02, d = 0.47] in the scaled equipment condition than full-sized condition.

Statistical parametric mapping found significant differences between variability in the upper arm and forearm angles close to impact in both the full-sized condition (t (1,21) = 2.99, p = 0.008) and the scaled condition (t (1,21) = 2.93, p = 0.03). In the full-sized condition, the difference between variability in the upper arm and forearm angles was trending linearly towards significance from mid-swing onwards. Conversely, in the scaled condition, the difference between variability in the upper arm and forearm angles only accentuated close to impact (see Supplementary Material).

**Figure 1:**
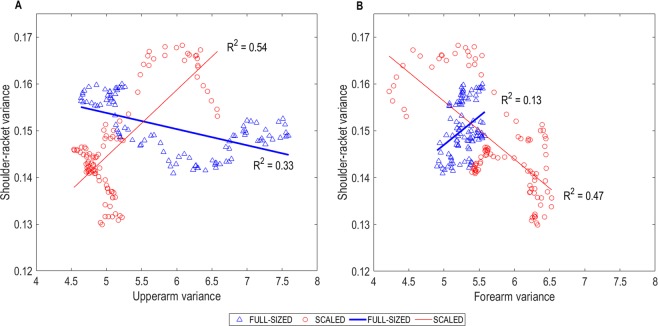
Correlation-coefficients (R^2^) between (**A**) upper arm variance and shoulder-racket distance variance, and (**B**) forearm variance and shoulder-racket distance variance for the two conditions.

**Figure 2:**
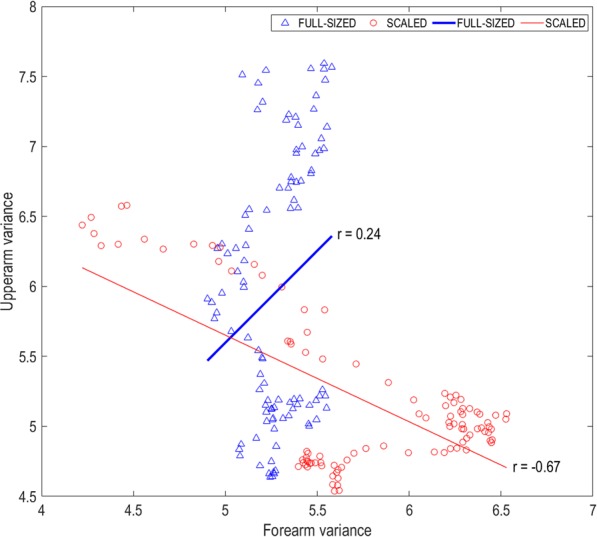
Correlation (r) between forearm and upper arm variance for the two conditions.

The racket-shoulder distance was regulated by forearm and upper arm angles in both conditions (R^2^ = 0.55, p < 0.01 in scaled condition, and R^2^ = 0.58, p < 0.01 in full-sized condition). In the scaled condition, variability in the forearm and upper arm angles largely explained variability in shoulder-racket distance (R^2^ = 0.47, p < 0.01 and R^2^ = 0.54, p < 0.01 respectively) (see Fig. 1A,B). Furthermore, variability in the forearm angle exhibited a significant negative correlation with variability in the upper arm angle (r = −0.67, p < 0.01) (see Fig. 2). Conversely, in the full-sized condition, variability in the upper arm angle moderately explained variability shoulder-racket distance (R^2^ = 0.33, p < 0.01), while variability in the forearm angle had a smaller contribution in explaining variability in the shoulder-racket distance (R^2^ = 0.13, p < 0.01). Moreover, the variabilities in the two segments were not significantly correlated with each other.

**Figure 3:**
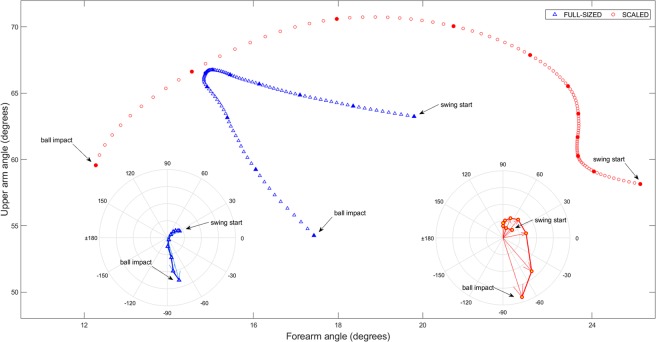
Relative motion plot for forearm and upper arm segment absolute angle. Data are from the mean of all trials executed in the full-sized and scaled conditions. Filled symbols represent the extremes of the ten calculated vectors. Polar plots at the bottom represent the inter-segmental coordination patterns for the two conditions.

**Figure 4:**
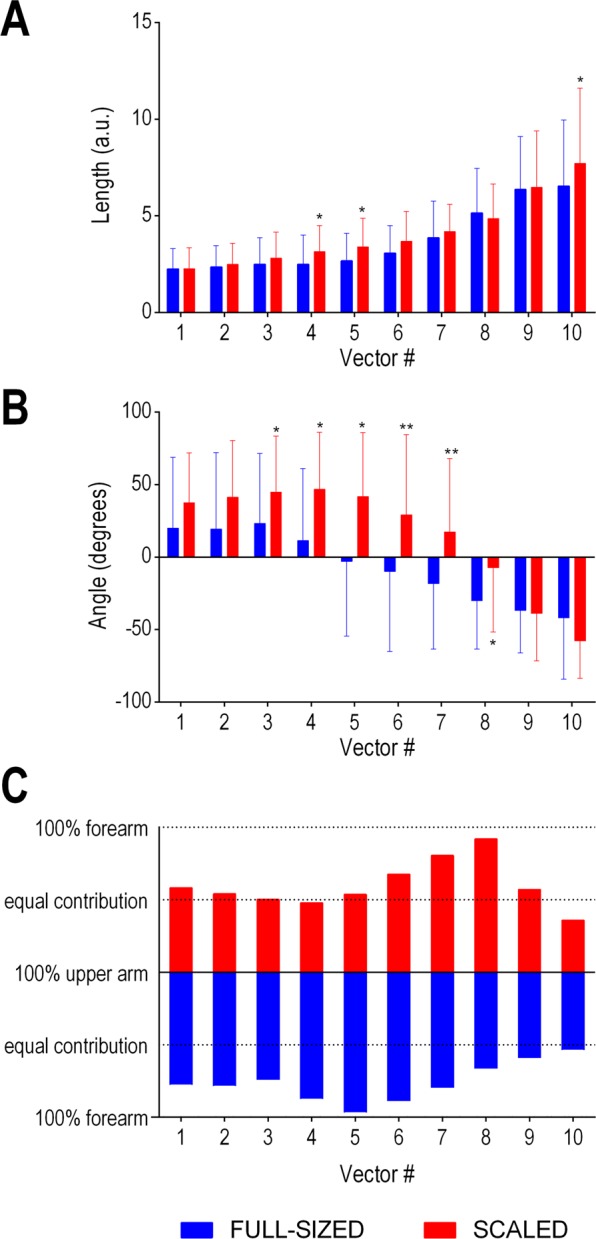
Coupling (**A**) lengths, (**B**) angles, and (**C**) segment angle contribution for the ten computed vectors are compared between conditions. *Represents statistically significant differences with *p* < 0.05; **p < 0.001.

Differences were also found between conditions on the coordinative pattern between upper arm and forearm (see Fig. 3). The swing movement in the scaled condition was faster (i.e., longer vectors) than the full-sized condition in vector 4 [t (21) = −2.74, p = 0.013, d = 0.47], vector 5 [t (21) = −3.00, p < 0.01, d = 0.50] and vector 10 [t (21) = −2.89, p < 0.01, d = 0.32] (see Fig. 4A). Significant differences were also found in absolute angle change in vector 3 [t (21) = −3.02, p < 0.01, d = 0.50], vector 4 [t (21) = −3.70, p < 0.01, d = 0.80], vector 5 [t (21) = −4.10, p < 0.01, d = 0.93], vector 6 [t (21) = −4.40, p < 0.01, d = 0.71], vector 7 [t (21) = −4.70, p < 0.01, d = 0.74], and vector 8 [t (21) = −3.45, p < 0.01, d = 0.59] (see Fig. 4B). The contribution to the change in trajectory of upper arm and forearm angles was balanced in most vectors (except vectors 6, 7, and 8) in the scaled condition, while the contribution of forearm angle was predominant in most vectors (except vectors 9 and 10) in the full-sized condition (see Fig. 4C).

## Discussion

The present study revealed contrasting patterns of change to movement variability and the coordination between segments of the hitting arm as a function of the equipment used in a tennis forehand task. Scaled equipment, which included a smaller racket and a lower compression ball, resulted in superior hitting accuracy and greater temporal stability of the swing compared to full-sized equipment. This suggests that scaled equipment promoted higher functionality of movement. Significantly, scaled equipment afforded the emergence of a functional coupling between the upper arm and forearm. Our results demonstrate how task constraints (i.e., equipment) affect children’s kinematics when performing a multi-articular action.

As expected, the hitting lever length – the distance between the shoulder and the racket – was regulated by the forearm and upper arm when using both scaled and full-sized equipment. Indeed, the regression analyses showed that the two segments explained a similar amount of variance in both conditions (55% and 58%). However, the manner in which the segments regulated this distance differed between the two conditions. When using scaled equipment, the shoulder-racket distance was regulated by the upper arm and forearm working in unison (i.e., coupled). Hence, movements at the intermediate elbow joint could move flexibly without altering the key parameter of the skill (i.e., shoulder-racket distance). Coupling body segments to functionally achieve a task goal is considered important for developing coordinated movements and therefore skill^22^. Comparatively, when using full-sized equipment, the shoulder-racket distance was regulated by the upper arm and forearm operating in a manner that lacked coupling. These differences were evident by four findings. First, both upper arm and forearm variance correlated with the shoulder-racket distance in the scaled condition, whereas only upper arm variance was significantly correlated with shoulder-racket distance variance in the full-sized condition (see Fig. 1A,B). Second, upper arm variance and forearm variance correlated with each other in the scaled condition, whereas no significant correlation was found in the full-sized condition (see Fig. 2). Third, there was minimal difference between upper-arm and forearm variability for most of the swing when using scaled equipment, whereas this difference was linearly increasing from mid swing onwards when using full-sized equipment (see Supplementary Material). Fourth, the upper arm trajectory and the forearm trajectory were tightly coupled when using scaled equipment, but not when using full-sized equipment (see Figs. 3 and 4C).

A closer inspection of the relationship between the upper arm and forearm highlighted significant differences in how the movement was controlled. When scaled equipment was used, the majority of the swing was controlled by an equal contribution from the upper arm and forearm (except for Vectors 7 and 8 in Fig. 4C). This further highlights the coupling between the segments. When full-sized equipment was used, however, the majority of the swing (i.e., the first 8 Vectors in Fig. 4C) was controlled mostly by the forearm. This indicates that participants froze degrees of freedom in the upper arm for most of the swing when using full-sized equipment. Indeed, the upper arm was typically only released just prior to ball impact. Notably, the switch between forearm and upper arm movements was abrupt, and was in stark contrast to the smooth movement associated with scaled equipment (see Fig. 3). The abrupt change with full-sized equipment indicates that the movements were more rigid, and this might also have contributed to the increased variability in swing time. Given that movements were also shorter and slower with full-sized equipment, it seems that children were fixating movements in the upper arm in an attempt to control mechanical degrees of freedom. By doing so, children’s ability to exploit mechanical degrees of freedom was limited. This has significant implications for skill acquisition.

Bernstein’s degrees of freedom problem asserts that skill acquisition progresses through stages^23^. Initially learners tend to rigidly fixate movements at the joints in attempt to control mechanical degrees of freedom. As skill develops, the learner releases mechanical degrees of freedom in an attempt to exploit abundant mechanical degrees of freedom via the co-variation between segments e.g.,^24^. The coupling between the upper arm and forearm observed when using scaled equipment in the current study draws parallels to the releasing of mechanical degrees of freedom. Additionally, co-variation between body segments is often accompanied by greater fluency and speed in the movements^21^. Scaled equipment promoted a smoother movement than full-sized equipment (see Fig. 3), and movement speed was greater during the middle phase of the stroke (see Fig. 4A). Our results suggest that appropriately sized equipment (i.e., scaled equipment) can facilitate skill acquisition. Comparatively, the freezing of the upper arm when using full-sized equipment was more akin to early stages of learning, and this begs the question of whether inappropriately sized equipment causes skill to regress to an earlier stage of learning (or, at least, hinder skill acquisition).

We do need to be mindful of at least two limitations in our study. First, our analysis focused on the upper-arm, forearm and racket, but neglected the hand and wrist. Although we intended to analyse hand and wrist data, unfortunately data quality issues presented by marker coalescence resulted in us electing to discard these data. Hand and wrist data may have added to our understanding of how the shoulder-racket distance was controlled during a forehand. A second limitation was that we did not capture ball trajectory and, therefore, we did not ascertain the variability in ball trajectory inherent to underarm throws (i.e. the ball feed). However, it is important to note that we opted to use an underarm throw rather than a ball-machine for reliability purposes. The accuracy of ball machines for standard tennis balls (i.e., the balls used in the full-sized condition) was reported to be 0.63 ± 0.34 m^25^, but from experience this error margin increases substantially when using lower compression balls (i.e., the balls used in the scaled condition). We therefore considered an underarm throw by the same skilled sports coach to a small target (1 × 1.5 m) to be a more reliable ball feed. As a consequence of not capturing ball trajectory, we also cannot exclude the possibility that children were attempting to strike the ball earlier or later in its trajectory in the full-sized condition as a result of the racket’s greater inertia and the higher rebound of the ball. This may cause a hurried or delayed action in contrast to the scaled condition.

The current study adds to the modified sport literature by demonstrating that scaled equipment can facilitate the emergence of functional movement variability whilst achieving temporal stability. More specifically, our data indicated that when children aged 6 to 9 years used full-sized equipment, movements were more likely to be controlled by the freezing of one segment (i.e., the upper arm in our study), whereas scaled equipment was more likely to promote movement that was controlled by the coupling of segments. An interesting question is whether appropriately sized equipment also facilitates coupling between perception and action e.g.^25^. Indeed, we speculate that the increase in forearm contribution closer to ball impact when using scaled equipment (see Fig. 4C) was due to children adapting the racket’s position to the ball’s location. This increase in forearm contribution closer to impact did not occur when using full-sized equipment. Hence, we suspect that scaled equipment allowed children to functionally adapt their movements to the dynamics of the ball^26^ (i.e., perception-action coupling). If we extrapolate our hypothesis to the development of interceptive skills such as striking a ball, scaled equipment should effectively constrain the performer-environment system to encourage the emergence of perception-action couplings important to the task. To verify our findings and interpretations, further research is required in which both hand and wrist kinematics and ball trajectory are collected in addition to the variables assessed in the current study. With this in mind, the examination of equipment scaling provides a rich avenue for investigating the effect of constraints on coordination, perception and action.

## Methods

### Participants

Twenty-five children (17 boys and 8 girls; *mean age* = 7.6 years ± 1.3; *mean height* = 128.9 cm ± 7.4) volunteered to participate in the study. All children were participating in Tennis Australia’s Hot Shots program at the time of testing. This is a junior modified tennis program and, therefore, all children had experience playing tennis using the equipment in our scaled condition. Children were not included if they had played tennis for more than two years or had played any competition tennis. Hence, children were considered beginners in tennis, but not novices. All children provided written assent to participate in the study while their parents provided written consent. The study was approved by the Research Ethics Committee at the university where the study was conducted.

### Protocol

Data collection took place at an indoor biomechanics laboratory, wherein children performed a forehand hitting task. The task required children to execute a forehand stroke with the aim of hitting the ball to a target located 10 metres away. The target was positioned directly in front of the children. The target represented a scoring system akin to a traditional bullseye target, but was arranged in squares. Balls landing in the smallest, central, “bullseye” square (0.5 × 0.5 m) were awarded 10 points. Target squares expanded outward from the central square in 0.5 m bands and scores iteratively decreased by 1 point as balls landed further from the “bullseye” (i.e., a score between 0 and 10 was given for every shot). The size of the court was in accordance with the International Tennis Federation’s recommendations for 6 to 8 year old children. Balls were fed by the experimenter (TB), who was standing next to the “bullseye” target - 10 metres away from the participant. The fed balls landed in a 1.0 m × 0.5 m box positioned 1.5 m from the net in the centre of the court. Any ball that landed outside of the box was discarded and the feed repeated. The maximum number of shots repeated in one condition due to an inaccurate feed for any participant was three. There was no significant difference between conditions in the total number of additional shots (*p* > 0.05). Participants also needed to remain within a designated hitting area whilst playing the shot. The designated hitting area was a defined by a 1.2 m × 1.2 m box positioned 0.5 m from the ball-landing box and on the forehand side (i.e., from the midline of the ball-landing box). Shots played from outside this area were repeated; however, this did not occur.

Participants performed the task in two conditions – a scaled equipment condition (referred to as the *scaled condition*) and a full-sized equipment condition (referred to as the *full-sized condition*). The scaled condition required participants to use a 21-inch racket and a low compression “red” ball (~25% compression of the standard yellow tennis ball). The full-sized condition required participants to use a 27-inch racket and a standard yellow tennis ball. The balls were different coloured to preserve ecological validity, as these colours are endorsed by the International Tennis Federation for these specific balls^27^. Participants performed three familiarisation shots in each condition before commencing. The conditions were performed in a counterbalanced order across participants, with 20 shots performed during each condition. A two-minute rest was provided between conditions.

### Data capture

**Figure 5:**
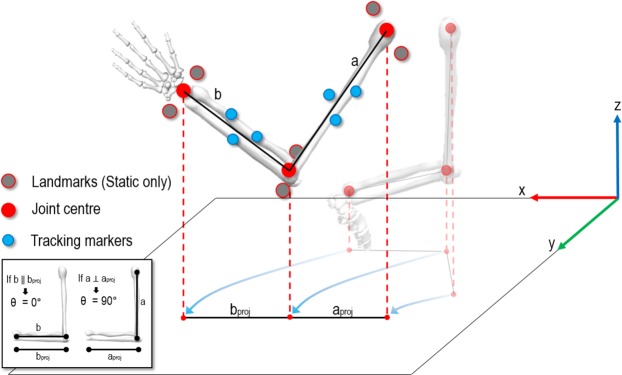
Biomechanical model of upper arm (**a**) and forearm. (**b**) Landmarks during the static trial were used to define joint centres, while tracking markers were used to compute segments’ position in space. By projecting arm segments onto the ground two new segments were created (a_proj_, b_proj_). The angles between actual segments (**a**,**b**) and projected segments (a_proj_, b_proj_) defined the segments’ angles.

Nine (14-mm) reflective markers were used to define coordinate systems of the hand, forearm and upper arm of the hitting limb. Nine markers were also placed over landmarks in a static trial to define the wrist, elbow and shoulder joint centres (Fig. 5). Four markers were used to model the racket: markers at the butt and tip defined the longitudinal axis and two either side of the racket head, the medio-lateral axis. Unfortunately, given that participants were children, there was limited surface area on the hand on which to place three distinct markers. Hence, hand marker coalescence presented a major challenge for the optical system, to the point where we did not feel comfortable imputing hand marker trajectories. As such, we elected to discard these data, precluding the possibility of reconstructing the position and orientation of the racket-hand system. Cartesian markers coordinates were recorded using a 100 Hz, 22-camera VICON MX system (VICON Motion Systems, Oxford, UK) and were smoothed using a Butterworth digital filter^28^, with a cut-off frequency of 15 Hz. In the global reference frame, positive x, y and z corresponding to right (parallel to the net), forward (toward the net) and upward, respectively.

The forward-swing was defined to commence when the position (in the y direction) of the racket changed from negative (going backward) to positive (going forward), while the impact frame was identified (using the raw – unfiltered data) at the nadir of y-acceleration of the racket tip marker. Transverse plane angles of the hitting arm’s segments (projected on to the ground) revealed their alignment relative to impact, with an angle of 0° represented a parallel position of the segment to the ground, while an angle of 90° a perpendicular position to the ground (see Fig. 5). This angular computation relies on projection of segment’s distal and proximal ends onto the ground, creating a constant plane on which the segment moves regardless its position in space. This method avoids misalignment error due to the multiplane motion of the segments during the swing movement^29^. Racket-shoulder distance was the Euclidean distance between the shoulder joint centre and racket tip marker. Kinematics for the left-handed players were inverted such that all data pertain to a right-hand dominant player.

### Variables of interest

Kinematic variables of interest included the variability in swing time, variability in racket-shoulder distance, and variability in forearm and upper arm angles. Variability in racket-shoulder distance, and forearm and upper arm angles were calculated between the initiation of the forward-swing and impact. Variability was computed using the MAD method^30^, which calculates the median of the differences between each data point and the calculated median across trials. The primary outcome variable was hitting accuracy, which was defined as the score (0 to 10) per shot.

### Statistical analysis

Differences in hitting accuracy and swing time variability between the scaled and full-sized conditions were tested for significance using a paired samples t-test. The waveforms of the angle variability (SD) in the upper arm and forearm were analysed using one-dimensional statistical parametric mapping (SPM). This method allows for evaluating changes (a) between the two conditions and (b) between upper arm and forearm variability within each condition across the whole swing motion. The effect of group or condition was analysed using a SPM two-tailed repeated measures t-test. The SPM t-test yielded a t-curve, or SPM(t), of which the significance was determined using random field theory^31^. Open source code for conducting SPM tests was obtained from https://www.spm1d.org and implemented in Matlab version R2018b.

Multiple linear regression models were used to determine how shoulder-racket distance variability was explained by variability in the upper arm and forearm angles in each condition. Furthermore, a Pearson correlation coefficient between upper arm and forearm angle variability was calculated in each condition.

An angle–angle plot described the relationship between the motion of the forearm and upper arm during the two conditions, while vector coding techniques^32^ quantified inter-segment co-variation through the swing. Swing time was normalized to 101 points and the angle-angle trajectory was divided into 10 vectors of equal time (i.e. 10 frames). The length of the vector represented movement velocity (i.e., longer vectors equals faster movements), whilst the vector’s angle represented the contribution of segments to the change in trajectory. This meant that 0° and ±180° represented a contribution of forearm angle only, an angle of ±90° represent a contribution of the upper arm angle only, hence a ±45° represent an equal contribution (see Fig. 4C). A paired sample t-test was used to test for differences in length and absolute angle of paired vectors. Statistical tests were conducted using SPSS (version 25.0. Armonk, NY: IBM Corp.); statistical significance was set at *p* < 0.05. The magnitude of differences was calculated using Cohen’s *d* and defined as follows: <0.2 trivial, 0.2–0.6 small, 0.6–1.2 moderate, 1.2–2.0 large, >2.0 very large, while the magnitude of correlations was defined as small (0.1), moderate (0.3), and large (0.5).

### Compliance with ethical standards

This study was carried out in Accordance with the recommendations of the National Statement on Ethical Conduct in Human Research (2007). All participants gave informed assent and written informed consent was provided by their parents or guardians in accordance with the National Statement. The protocol was approved by the Victoria University Human Research Ethics Committee.

## Supplementary information

Supplementary information

## Data Availability

The datasets generated during and/or analysed during the current study are available from the corresponding author on reasonable request.
