# Supplementary material for: Scaling sports equipment for children promotes functional movement variability
Source: Sci Rep. 2020 Feb 20;10:3111. doi: 10.1038/s41598-020-59475-5 (PMC7033277; doi:10.1038/s41598-020-59475-5)
Supplement: Supplementary file 1 — Supplementary information [file 41598_2020_59475_MOESM1_ESM.docx]

# Scaling sports equipment for children promotes functional movement variability

Tim Buszard^#,^*^,^, Alessandro Garofilini^#^, Machar Reid, Damian Farrow, Luca Oppici, David Whiteside

#Equal Contribution as First Authors

*Corresponding Author

Correspondence to tim.buszard@vu.edu.au


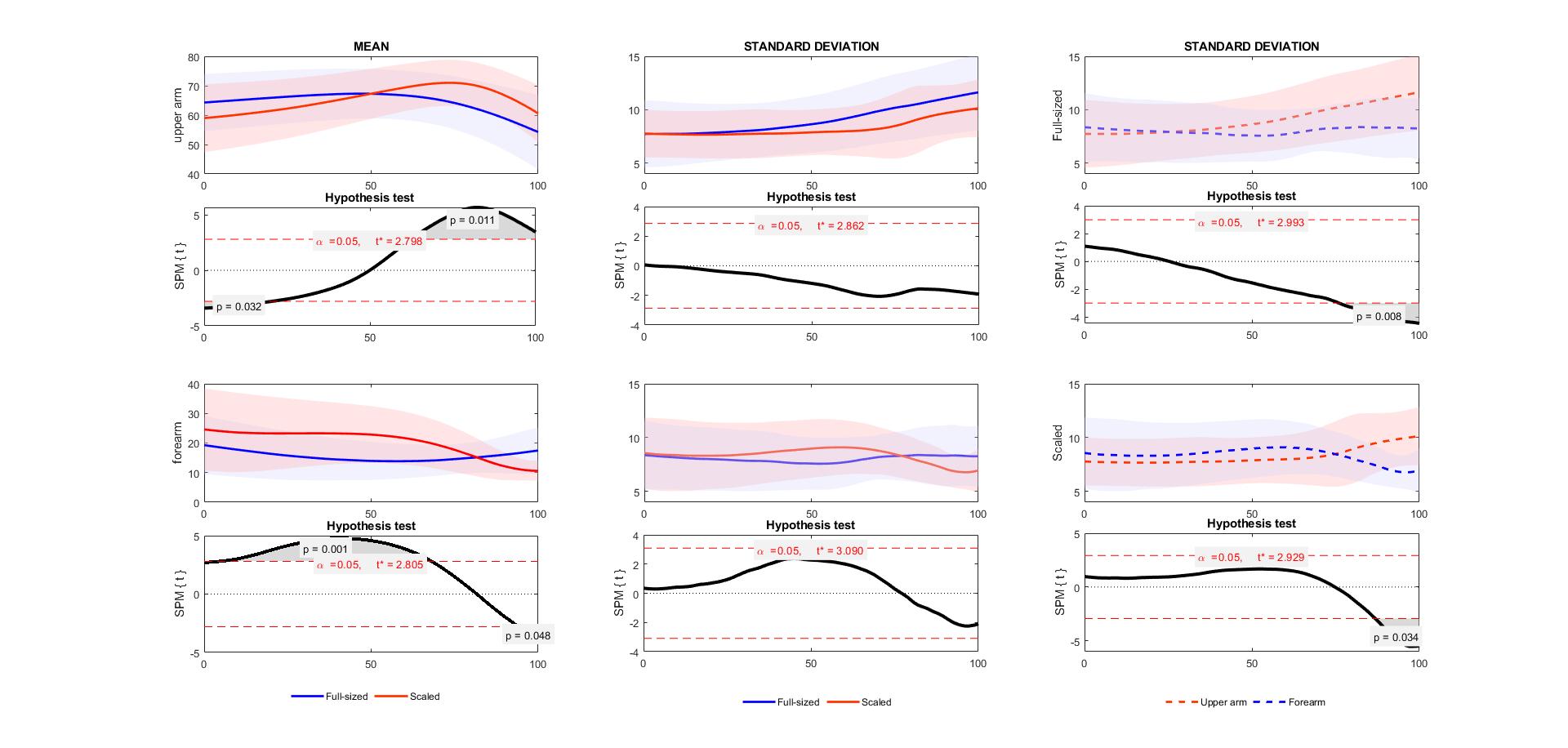


Supplementary Figure. Results from the one-dimensional statistical parametric mapping analysis which assessed the waveforms of angle variability (SD) in the upper arm and forearm between and within the two conditions.
